# Planning and management to control and eliminate HTLV-1 infection in Iran

**DOI:** 10.22038/ijbms.2021.50803.11562

**Published:** 2021-03

**Authors:** Reza Boostani, Nasim Lotfinejad, Fariba Zemorshidi, Zohreh Vahidi, Seyed Abdolrahim Rezaee, Reza Farid, Houshang Rafatpanah

**Affiliations:** 1 Department of Neurology, Mashhad University of Medical Sciences, Mashhad, Iran; 2 Department of Research, Faculty of Medicine, Mashhad University of Medical Sciences, Mashhad, Iran; 3 Immunology Research Centre, Inflammation and Inflammatory Diseases Division, Mashhad University of Medical Sciences, Mashhad, Iran; 4 Allergy Research Centre, Mashhad University of Medical Sciences, Mashhad, Iran

**Keywords:** ATL, HAM/TSP, HTLV-1, Prevention, Treatments

## Abstract

Prevention and treatment of the Human T-cell leukemia virus, type 1 (HTLV-1) which was discovered nearly 40 years ago, still remain challenging. The reported high prevalence of HTLV-1 in some countries around the world triggered an open letter to the World Health Organization (WHO), urging action against HTLV-1 infection in 2018. This highlights the importance of virus elimination strategies to eradicate HTLV-1 infection. In Iran, we have documented our experiences with the virus in order to achieve and promote the possible ways to manage, control, and eliminate HTLV-1. Although there has been considerable progress apropos of HTLV-1, a series of additional challenges need to be tackled to control HTLV-1 infection in Iran.

## Introduction

Since the discovery of the human T-cell leukemia virus, type 1 (HTLV-1) nearly 40 years ago, prevention and treatment of the virus’ two main associated diseases, HTLV-1-associated myelopathy/tropical spastic paraparesis (HAM/TSP) and adult T-cell leukemia (ATL) still remain challenging (1, 2). An open letter to the World Health Organization (WHO) by Martin *et al*. has highlighted the need for urgent action in order to eradicate HTLV-1 infection and subsequently virus elimination strategies have been reported from Japan (3, 4). The government measures established against HTLV-1 in Japan are routine HTLV-1 testing as a part of antenatal pregnancy screening, counseling for patients and training healthcare workers, improving the coordination of care for HTLV-1-associated diseases, updating a website and developing communication materials for the public, and promoting research about HTLV-1-associated diseases (4).

In line with HTLV-1 eradication plans, we have documented our experiences with the virus in order to achieve and promote the possible ways to manage, control, and eliminate HTLV-1. 

HTLV-1 infection is highly concentrated in the northeast of Iran, with a significant prevalence in the cities of Neyshabur (3-5%), Mashhad (2.1%), and Sabzevar (1.66%) (5, 6). However, the virus has also spread to other parts of Iran such as Alborz (centre), Ardabil (northwest), Gilan (north), and West Azerbaijan (7-9). 

The success of any disease eradication initiative can be primarily evaluated by focusing on endemic regions where the disease is most prevalent (10). Therefore, in the past few years, Iran has launched an unprecedented effort to control HTLV-1 infection in the immediate vicinity of these regions. Based on the WHO recommendations about screening all blood donations for HTLV-1 in endemic areas, the first strategy of the Iranian Blood Transfusion Organization (IBTO) was to begin this screening process for HTLV-1 in the northeast of Iran, beginning in 1995. This policy was further expanded and nowadays HTLV-1 antibody is regularly screened at blood transfusion centres of 7 out of 31 provinces (7). Moreover, additional strategic plans have been put into place for prevention of HTLV-1 transmission in Mashhad, Neyshabur, and Sabzevar that include use of routine screening to stop mother-to-child transmission for HTLV-1 in pregnancy and screening of hemodialysis patients for HTLV-1. 

In the process, HTLV-1 Foundation has been established by Mashhad University of Medical Sciences (MUMS) as a national network at Ghaem hospital since 2011 with the aim of increasing public awareness (http://www.htlv-1.ir/). A variety of experts in the fields of neurology, haematology, immunology, and microbiology all make up members of the HTLV-1 Foundation alongside representatives of blood transfusion organizations and the vice chancellors for research and health of MUMS. 

History has proved that the goals of eradication programs are not achieved, unless they are supported by precise assessment and data analysis. Consequently, we launched a national program to establish the first national HTLV-1 registry in Iran at Ghaem Hospital, MUMS, since 2017. This hospital-based registry, a first in the Middle East region, is considered a major step forward in managing asymptomatic carriers (ACs) and patients with HTLV-1. Ethics approval was obtained from the Biomedical Ethics Committee of MUMS and Ministry of Health and Medical Education, Iran. Registered patients and ACs are mostly referred from the Blood Transfusion Centre, health centres, and outpatient clinics in accordance with a suspicious positive HTLV-1 screening result. These patients are further assessed by enzyme-linked immunosorbent assay (ELISA) and their diagnosis is confirmed via a polymerase chain reaction (PCR) or Western blot tests. Among them, HTLV-1 patients, which show clinical symptoms, are stratified as HAM/TSP or ATL, whilst ACs are considered as HTLV-1 carriers in our registry. Currently patients diagnosed with ATL referred to us directly by hematologists are not included in our registry; however plans are in place to rectify this soon.

Our aim of expanding this registry throughout the entire population of Iran began with assembling a data collection team that included trained experts designed to work under supervision of two neurologists and an immunologist, working closely with Mashhad Neurology department. We have carefully and continuously collected demographic, clinical and laboratory (including DNA and RNA) data since 2018 of 100 HAM/TSP patients and 120 ACs in an electronic database. Prior to data collection and blood sampling, our patients signed an informed consent. The confidentiality of these data are maintained, and all the patients sign an informed consent form for having their data gathered and tested.

Patients are visited by our neurologist, and history and physical examinations are documented, diagnostic tests are performed free of charge, and the treatment course is followed routinely. The follow-up of the ACs is performed on an annual basis, whereas HAM/TSP patients are followed every six months and the progressive type of HAM/TSP is followed every three months. In our outpatient clinic on every Wednesday, we provide counselling services in addition to visiting the patients and regularly evaluating their treatment program. 

The HTLV-1 registry program has enabled us to monitor and improve our care for the patients as well as creating a platform for us to conduct better quality research. MUMS annually spends 2 billion Rials (approximately US$ 8,000) to perform research in the field of HTLV-1-associated diseases. So far, about 7 clinical trials have been designed in our registry program regarding HTLV-1 infection (11, 12). In point of fact, such databases are fundamental in creating a worldwide network for the purpose of achieving therapeutic guidelines and ultimately eradication of HTLV-1.

Sweeping efforts have been implemented to inform and educate the Iranian population regarding the HTLV-1 virus. In 2009 for the first time, an international congress of HTLV-1 and associated diseases was held in Mashhad. The following year in 2010, a range of informative books and a website were prepared by MUMS to provide general information about HTLV-1 in order to improve social awareness. Furthermore, since 2007, around 10 retraining programs have taken place for health-care workers mainly in the endemic regions of Mashhad and Neyshabur. To contribute on this expanding knowledge on HTLV-1, we plan to collect roughly 10000 samples from a cohort study amongst faculty and staff members of MUMS, allowing us to screen for the HTLV-1 virus and be able to conduct relevant research. 

Although there has been a considerable progress apropos of HTLV-1, a series of additional challenges need to be tackled to control and eliminate HTLV-1 infection in Iran. Expanding the aforementioned strategies throughout Iran, with its many endemic regions, will help to control and eliminate HTLV-1 in our country. We will continue at every level in the path towards this goal by ensuring high quality clinical data is available for future research. 
